# Uncoupling of Natural IgE Production and CD23 Surface Expression Levels

**DOI:** 10.1371/journal.pone.0062851

**Published:** 2013-04-30

**Authors:** Weicheng Ren, Kristina Lagerstedt, Ola Grimsholm, Anna Stern, Jia-Bin Sun, Yu Fang, Zou Xiang, Inga-Lill Mårtensson

**Affiliations:** 1 Department of Rheumatology and Inflammation Research, University of Gothenburg, Gothenburg, Sweden; 2 Department of Microbiology and Immunology, University of Gothenburg, Gothenburg, Sweden; 3 Mucosal Immunobiology and Vaccine Center, University of Gothenburg, Gothenburg, Sweden; 4 Department of Microbiology and Immunology, Affiliated Hospital of Guiyang Medical College, Guiyang, China; Université Libre de Bruxelles, Belgium

## Abstract

CD23, the low affinity receptor for immunoglobulin E (IgE), has been proposed to play a critical role in the regulation of IgE production, based on altered IgE levels in CD23-deficient mice and transgenic mouse models, as well as in mouse strains with mutations in the CD23 gene, e.g. 129 substrains. Here, we have investigated a mouse line termed LxT1 that expresses reduced CD23 surface levels on B cells, and its influence on natural IgE production. Extensive phenotypic analysis showed that CD23 surface expression was reduced in LxT1 compared to the control, without affecting B cell development in general. This CD23^low^ surface level in LxT1 mice is not as a result of reduced CD23 mRNA expression levels or intracellular accumulation, but linked to a recessive locus, a 129-derived region spanning 28 Mb on chromosome 8, which includes the CD23 gene. Sequence analysis confirmed five mutations within the CD23 coding region in LxT1 mice, the same as those present in New Zealand Black (NZB) and 129 mice. However, this CD23^low^ phenotype was not observed in all 129 substrains despite carrying these same CD23 mutations in the coding region. Moreover, serum IgE levels in LxT1 mice are as low as those in the C57BL/6 (B6) strain, and much lower than those in 129 substrains. These data indicate that the CD23 surface level and serum IgE level are uncoupled and that neither is directly regulated by the mutations within the CD23 coding region. This study suggests that caution should be taken when interpreting the immunological data derived from mice with different genetic background, especially if the gene of interest is thought to influence CD23 surface expression or serum IgE level.

## Introduction

CD23, the low affinity receptor for immunoglobulin E (IgE), is a type II transmembrane protein and a member of the C-type lectin family [1]. In terms of its structural features, CD23 is composed of a carboxy-terminal lectin domain, a stalk region, a transmembrane domain and a short, cytoplasmic tail. In mice, CD23 has been detected on the surface of B cells [2], follicular dendritic cells [3] and the gut epithelium [4]. For analysis of B cell subpopulations by flow cytometry, CD23 is widely used in combination with other markers to differentiate between subsets of immature, transitional (T) B cells designated T1 (IgM^hi^CD23^−^), T2 (IgM^hi^CD23^+^) and T3 (IgM^low^CD23^+^), and mature, follicular (FO) (IgM^low^CD21^hi^CD23^hi^) and marginal zone (MZ) (IgM^hi^CD21^hi^23^low/−^) B cells [5]–[7].

It has been proposed that CD23 plays an important role in the negative feedback of IgE production *in vivo* [8]. This was based on increased IgE levels in CD23 deficient mice [8] as well as after anti-CD23 antibody treatment of normal mice [9], in addition to the decreased IgE levels in CD23 overexpressing mice [10]. Given the significance of CD23 in IgE regulation, mutations in CD23 could potentially impair its surface expression and perturb its function. The New Zealand Black (NZB) and 129/SvJ strains carry the same five mutations in the CD23 coding region and have been reported to express reduced CD23 surface levels and hyper IgE serum levels [11], [12]. Out of the five mutations, three are located in the stalk region and two in the lectin domain [12]. The mutations within the stalk region might interrupt the trimeric structure of CD23 [11], [12], while the mutations in the lectin domain could interfere with its ability to bind IgE [13]. Studies in NZB mice suggested that the mutations could interfere with the trimerisation of CD23 and, in addition, suggested a dominant effect on CD23 surface expression and IgE levels [11]. Reduced CD23 surface levels have also been reported for the 129/SvJ [12], but not the 129/Sv substrain [14]. Since 129 strains are frequently used for gene targeting studies [15] and they all carry the same five mutations in the CD23 gene [12], mouse models on a mixed 129 genetic background may retain these mutations on either one or both alleles, and interfere with data interpretation.

In this study, we set out to investigate the relationship between reduced CD23 surface expression and natural IgE levels in a mouse line on a mixed C57BL/6 (B6)/129 background established in the lab. This line was termed LxT1, as it presented with what appeared to be a “block” at the transition from the T1 to T2 cell stage during B cell development, based on the reduced CD23 surface expression on B cells. We find that this CD23^low^ phenotype is associated with a recessive locus in a 28 Mb region of chromosome 8. This region includes the CD23 gene and carries the five mutations within the CD23 coding region found in NZB and 129 mice. Although both LxT1 and 129 mice share the same CD23 mutations in the coding region, they show different levels of CD23 surface expression and natural IgE production compared to the control. These data suggest that CD23 surface levels and natural serum IgE levels are not linked, and that the levels of CD23 surface expression and serum IgE levels are not directly regulated by the mutations in the CD23 gene.

## Materials and Methods

### Ethics Statement

All animal experiments were carried out with the approval of the Ethical Committee for Laboratory Animals in Gothenburg (permit numbers 245/09 and 39/10). The mice were bred and maintained in the Laboratory for Experimental Biomedicine facility at University of Gothenburg.

### Mice

C57BL/6 (B6) and BALB/c mice were purchased from Charles River (Germany), B6;129S4-Fcgr2b^tm1Ttk^/J (FcγRIIB^−/−^) (stock: 002848), 129S4/SvJaeJ (stock: 009104) and 129S1/SvImJ (stock: 002448) were from the Jackson Laboratory (Bar Harbor, Marine, US). All mice were analyzed at an age of two to four months. For analysis of IgE levels, the 129S4/SvJaeJ and 129S1/SvImJ mice were nine months, LxT1 and C57BL/6 mice were from three to nine months old.

Generation of LxT1 mice: FcγRIIB^−/−^ mice expressing CD23^low^ surface levels were crossed to B6 mice and then followed by intercrossing offspring to establish FcγRIIB^+/+^ mice with a CD23^low^ phenotype, hence independent of FcγRIIB. This line was named LxT1 as it presented with what appeared as a “block” at the T1 to T2 cell stage transition during B cell development, based on the surface expression of CD23.

The 129 substrains have changed nomenclature over the years as follows, current (previous): 129X1/SvJ (129/SvJ, 129X1), 129S2SvPasCrl (129/Sv), 129S1/SvImJ (129/SvImJ), 129S3/SvIm, (129S3/SvImJ), 129S4/SvJaeJ (129S4/SvJae). For a complete history of the numerous 129 substrains, see Simpson et al. 1997 [16].

### Flow Cytometry

Spleen, bone marrow, blood (after red blood cell lysis) and lymph node cell suspensions were stained with antibodies recognizing CD45R/B220 (RA36B2), CD93 (AA4.1) (eBioscience), CD19 (1D3), CD21 (7G6), CD23 (B3B4), IgD (IA6-2), CD32 (D34–485) (BD), CD23 (2G8) (Southern Biotech) and polyclonal goat anti-mouse IgM (Sigma). For intracellular staining of CD23, spleen cells were first stained with CD19 and CD23 (B3B4) in PE, then fixed and permeabilized according to the manufacturers instructions (Fix and Perm, eBiosciences), followed by staining for intracellular CD23 (B3B4) in PE-Cy7. To measure the total CD23 protein pool, CD23-PE was used in both steps. Cells were acquired on a FACS Canto II or a LSR-II (BD Biosciences) and data was analyzed using FlowJo software (Treestar Inc).

### Genetic Mapping and Linkage Analysis

To map a potential mutation, male LxT1 mice were crossed to female BALB/c mice to generate F1 mice. Two females from the F1 litters were backcrossed to the parental male LxT1 to generate N2 mice for analysis of phenotype and genetic mapping. N2 mice with (n = 6) and without (n = 5) CD23^low^ surface level and (n = 1) BALB/c were used to map putative genetic alteration that may cause the CD23^low^ phenotype. DNA was isolated from tails and ears using the Purgene core kit (Qiagen, Valencia, CA). The genotyping was performed at Aros Applied Biotechnology (Aarhus, Denmark) using Affymetrix Mouse Diversity Genotyping Array. The array can interrogate more than 623,000 single nucleotide polymorphisms (SNPs) derived from 12 classical inbred and 7 wild mouse strains, yielding an average SNP density of one marker per 4.3 kb [17]. SNP calls were generated by using Affymetrix scanner 3000 7G together with Affymetrix Genotyping Console Software version 4.0. Genetic linkage analysis was performed to exploit the co-segregation of chromosomal regions with the CD23 phenotype to identify the location of causative genes. The linkage analysis was conducted by homozygosity mapping (recessive mode of inheritance) using a custom R script. In the analysis, only SNP markers with calls in all mice and a call-confidence lower than 0.1 were considered. The data has been submitted to Gene Expression Omnibus and the accession number is GSE44231.

### RNA Isolation and Analysis

Spleen B cells were purified using CD19 MACS beads according to the manufacturer’s instructions (Miltenyi Biotec). Total RNA was isolated using Trizol (Invitrogen) as recommended by the manufacturer. Reverse Transcript II (Invitrogen) was used to make cDNA according to the manufacturer’s instructions. CD23 and ADAM10 mRNA levels were quantified by q-PCR and normalized to the expression of 18S rRNA. Primers and TaqMan probes: CD23 and 18S rRNA [18], ADAM10 (Mm00545742_m1, Applied Biosystems). Q-PCR was performed using ViiA7 and analyzed with the ViiA7 basic software (Applied Biosystems).

For sequencing of the CD23 coding region in LxT1 mice, two primer sets (TGG GAA CCT CCT AGA AAG CGT and TCC ACA GCT TTG CCA CCT CT, AGC GCA CAG CCT CCG ATT CT and GGG GTG GGC CTT GTT GGA GT) were designed using www.ncbi.nlm.nih.gov/tools/primer-blast. The following cycles were used: 95°C for 1 min, 35 cycles of 95°C, 30 s; 60°C, 30 s; 72°C, 30 s, and then 72°C, 5 min. The products were cloned into the pGEM-T easy vector (Promega) and sequenced at Eurofins MWG Operon (Germany).

### IgE ELISA

Serum IgE levels were measured by ELISA using plates coated with 1 µg/ml of the capture antibody (Rat anti-mouse IgE, R35–92). Standard curves were generated with purified mouse IgE (C38-2) and IgE levels were quantified by biotinylated anti-mouse IgE (R35–118) followed by streptavidin-Horseradish Peroxidase (GE Healthcare, UK). Plates were developed using tetramethylbenzidine (BD) and read at a wavelength of 450 nm using a microplate reader Spectra Max340PC (Molecular Devices, US). Values were analyzed based on a five-parameter analysis for the standard curve.

### Immunohistochemistry

Spleens were harvested from LxT1 and B6 mice and embedded into OCT compound (TissueTek, Tokyo, Japan), snap-frozen in liquid nitrogen and stored at −80°C until use. Tissues were cut into 8 µm thick sections using a cryostat (Leica, Wetzlar, Germany), and transferred to slides for staining, as previously described [19], using goat anti-mouse IgM-Texas red and IgD-biotin (11–26) (Southern Biotech) followed by SA-Alexa 488 (Invitrogen). Images were acquired using LSM 700 confocal microscope and ZEN 2009 acquisition software (Zeiss, Germany).

### Statistics

P-values were calculated using the two-tailed Student’s t-test (GraphPad Prism 6). *, p<0.05; **, p<0.01; ***, p<0.001, ****, p<0.0001.

## Results

### CD23 Surface Expression is Reduced on LxT1 B Cells

**Figure 1:**
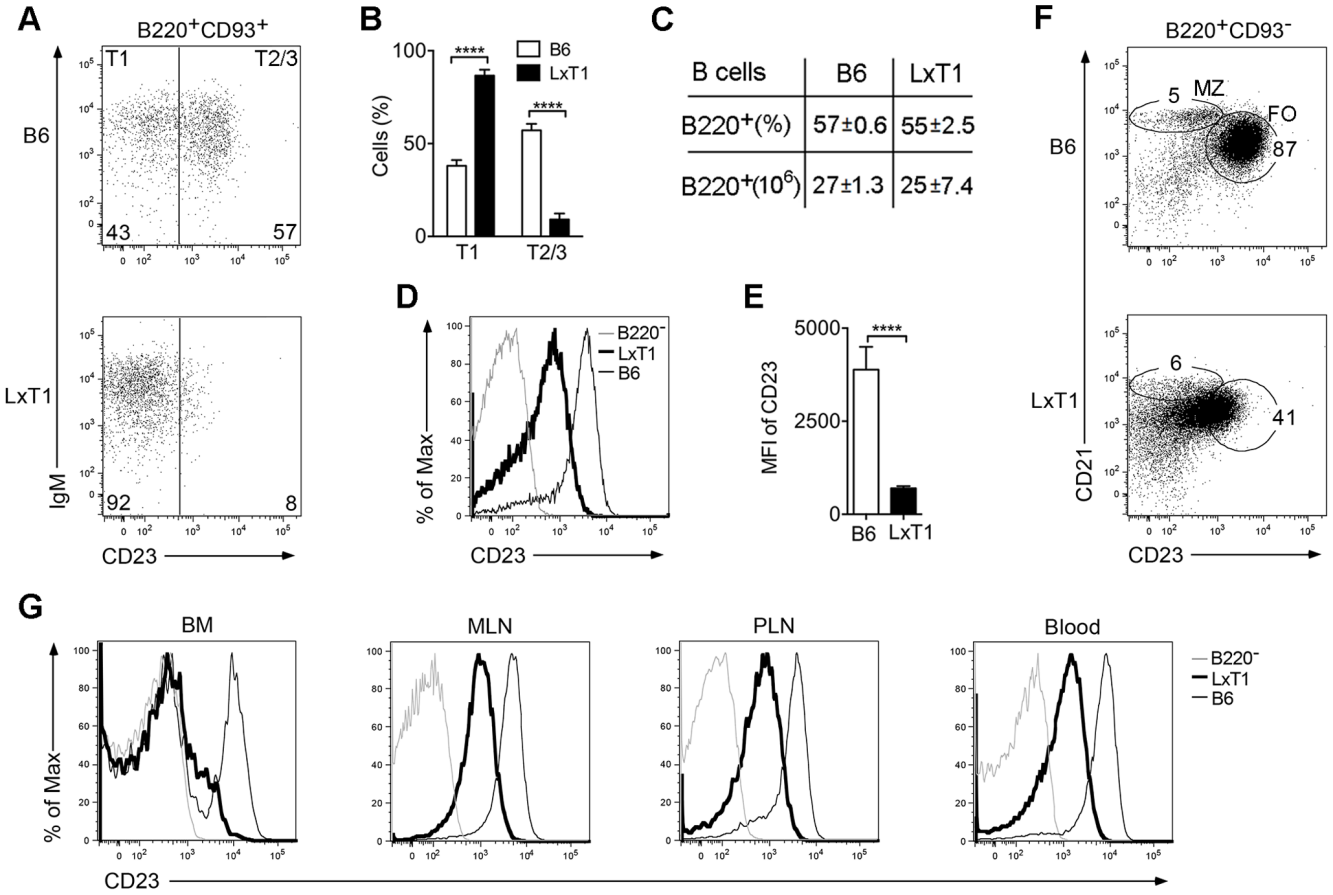
Reduced CD23 surface expression on LxT1 B cells. Flow cytometry analysis of spleen cells from two to three months old B6 and LxT1 mice. In LxT1 mice, the distribution of transitional (B220^+^CD93^+^) B cells shows an increased proportion of T1 and a reduction in T2/T3 (A and B). This is not as a result of a block in B cell development, as B cells still develop with numbers similar to those in B6 mice (C), but due to reduced CD23 (CD23^low^) surface levels on LxT1 B cells (D and E), resulting in a skewed distribution of mature (B220^+^CD93^−^) marginal zone (MZ) and follicular (FO) B cells (F). (G) The CD23^low^ phenotype is not unique to splenic B cells as it is found also on B cells from other lymphoid organs, i.e. bone marrow (BM), mesenteric and peripheral lymph nodes (MLN, PLN) and blood. (A-G) representative data from one experiment, out of three (n = 3/genotype/exp).

In the course of intercrossing various mouse lines to analyze their composition of B cell populations, we established one line termed LxT1, in which the transitional (B220^+^CD93^+^) T1 B cell population was increased and the T2/T3 population decreased (Fig. 1A and 1B). This was not due to a block in B cell development, as mature B cells still developed and B cell numbers were comparable to those in B6 mice (Fig. 1C), but rather as a result of the reduced CD23 (CD23^low^) surface levels on LxT1 B cells (Fig. 1D and 1E). To determine whether these results were due to different CD23 clones, another monoclonal antibody (2G8) was tested. Using this, CD23^low^ surface levels were also observed when comparing LxT1 and B6 spleen B cells (data not shown). The CD23^low^ phenotype made it difficult to determine the distribution of mature (B220^+^CD93^−^) FO and MZ B cells when employing the widely used CD23 in combination with CD21 [7], as evident when comparing with B6 mice (Fig. 1F). Moreover, the CD23^low^ phenotype in LxT1 mice was not unique to splenic B cells, as CD23^low^ surface levels were observed also on B cells from other lymphoid organs, i.e. bone marrow, lymph nodes and blood (Fig. 1G).

### The Splenic B Cell Compartment is Normal in LxT1 Mice

**Figure 2:**
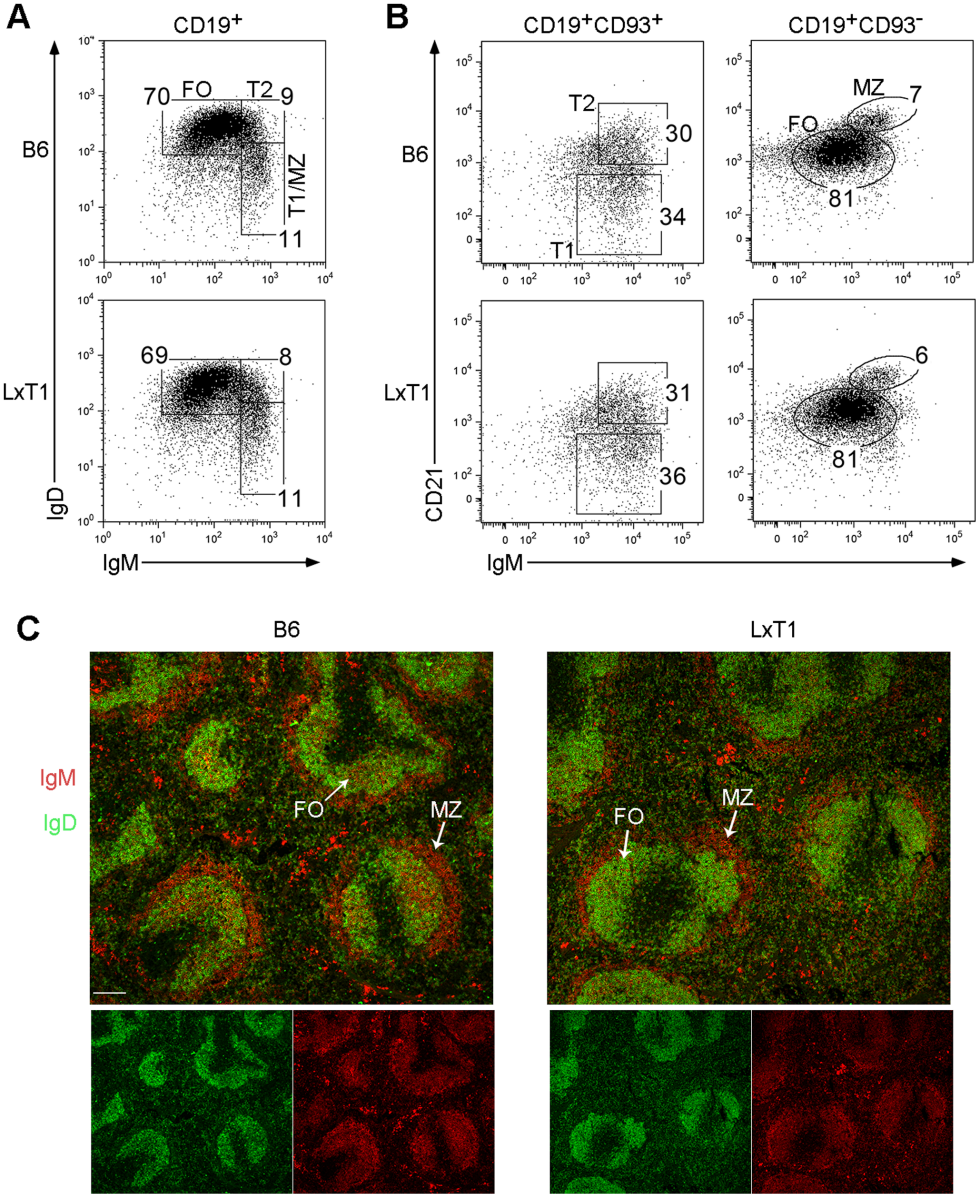
Normal distribution of B cell populations in LxT1 mice. Flow cytometry analysis of spleen cells from two to four months old B6 and LxT1 mice. Compared to B6, LxT1 mice show normal development of B cell populations based on the expression of IgM vs IgD, gated on CD19^+^ cells (A) and IgM vs CD21 (B), gated on CD19^+^ with/without CD93. (A) T1/MZ, (IgM^hi^IgD^low^); T2, (IgM^hi^IgD^hi^); FO (IgM^low^IgD^hi^). (B) T1 (CD93^+^IgM^hi^CD21^−^); T2 (CD93^+^IgM^hi^CD21^+^); FO (CD93^−^IgM^low^CD21^int^); MZ (CD93^−^IgM^hi^CD21^hi^). Representative data from one out of three experiments. (C) Immunofluorescence staining of spleen cryosections from LxT1 (n = 6) and B6 mice (n = 3) shows normal localization of FO and MZ B cells after staining for IgM (red) and IgD (green). 10× objective, bar, 100 µm.

Because CD23 surface levels have been implicated in regulating IgE production [9], we investigated the CD23^low^ phenotype further. We first analyzed the splenic B cell compartment in LxT1 mice, which showed that the levels of IgM and IgD on CD19^+^ cells were similar comparing LxT1 with B6 mice (Fig. 2A). Defining different B cell populations using these markers demonstrated that the percentages of T1/MZ, (IgM^hi^IgD^low^), T2, (IgM^hi^IgD^hi^) and FO (IgM^low^IgD^hi^) populations were similar in LxT1 and B6 mice (Fig. 2A), suggesting that the development of these B cell populations is normal. To investigate this further, CD21 and CD93, two markers utilized to define different B cell populations [5], [7], [20], [21], were also included. Gating CD19^+^ B cells and comparing those from LxT1 with B6 mice showed that the proportions of T1 (CD93^+^ IgM^hi^CD21^−^) and T2 (CD93^+^IgM^hi^CD21^+^), as well as those of FO (CD93^−^IgM^low^CD21^int^) and MZ (CD93^−^IgM^hi^CD21^hi^) cell populations were also similar (Fig. 2B). Fluorescence microscopy of splenic cryosections demonstrated that the localization of FO and MZ B cell populations were normal upon comparing LxT1 and B6 mice (Fig. 2C). Taken together, the levels of CD23 surface expression was reduced on LxT1 B cells, rather than the development of different B cell populations in spleen.

### LxT1 and B6 Mice Display Similar Serum IgE Levels

**Figure 3:**
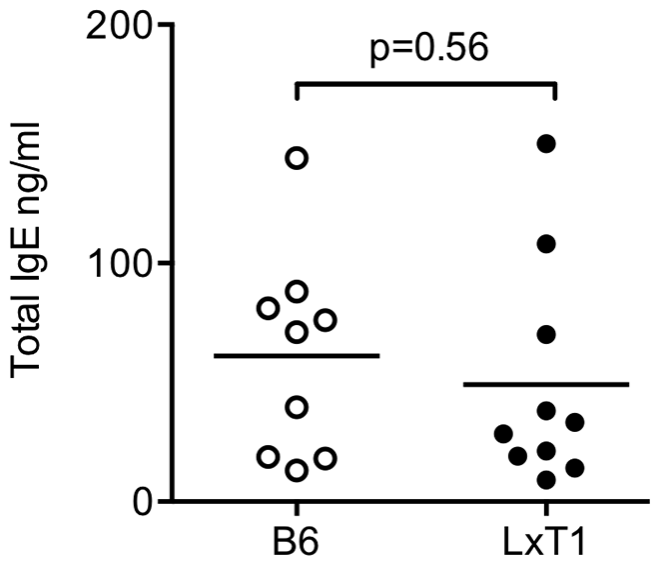
LxT1 mice display normal natural IgE levels. Similar serum IgE levels in LxT1 and B6 mice in the absence of active immunization. Each symbol represents an individual mouse. Black bar represents mean values. Representative of 2 experiments.

Previous studies of various mouse models suggested that CD23 surface levels regulate IgE production *in vivo*, with low surface levels linked to high serum IgE levels and *vice versa* [9]. To determine whether the CD23^low^ surface level was reflected in high natural IgE production in LxT1 mice, serum IgE levels were measured. Unexpectedly, we found that LxT1 and B6 mice displayed similarly low IgE levels (Fig. 3). Because the LxT1 mice presented with reduced CD23 surface levels, these results would argue against a direct link between natural IgE production and CD23 surface levels on B cells.

### LxT1 Mice Display Mutations in the CD23 Coding Region

Previous studies have implicated five mutations in the CD23 coding region that are correlated to reduced CD23 surface expression and hyper IgE levels [11], [12]. This lead us to consider whether the CD23 mutations could be responsible for the CD23^low^ phenotype observed on LxT1 B cells. Sequencing of the CD23 coding region from LxT1 splenocytes revealed five mutations as compared to the B6 and BALB/c strains (Table 1), in fact the same mutations as those reported for the 129/SvJ and NZB strains [12]. Further examination of published SNPs in the CD23 coding region from other mouse strains was carried out based on the literature and CGDSNP database with more than 66 million SNPs from more than 100 strains of laboratory mice (http://cgd.jax.org/cgdsnpdb/) [22]. The results revealed that the five mutations in the CD23 coding region are also present in other strains, including NZB, 129P1/ReJ, 129X1/SvJ, 129S1/SvImJ (129S1) and 129S4/SvJaeJ (129S4) (Table 1). Each of these five mutations leads to amino acid substitutions, some of which that could potentially disturb the CD23 structure [12]. Therefore, it appears as if the CD23^low^ surface levels on LxT1 B cells might be associated with these five mutations.

**Table 1:** Mutations in the CD23 coding region in different mouse strains.

Strains	rs32875516	rs3690909	rs32835920	rs32640440	rs32785864
C57BL/6J	G	T	A	C	G
BALB/cJ	G	T	A	C	G
129X1/SvJ	**A**	**C**	**G**	**T**	**A**
129P1/ReJ	**A**	**C**	**G**	**T**	**A**
129S1/SvImJ	**A**	**C**	**G**	**T**	**A**
129S4/SvJaeJ	**A**	**C**	**G**	**T**	**A**
NZB/BinJ	**A**	**C**	**G**	**T**	**A**
NZW/LacJ	G	T	G	C	G
LxT1	A	C	G	T	A

CD23 mutations (bold) are reported in the CGDSNP database and literature [12]. The mutations in CD23 coding region in LxT1 are demonstrated by sequencing.

### Lack of CD23^low^ Phenotype but Presence of Hyper IgE in 129 Substrains

**Figure 4:**
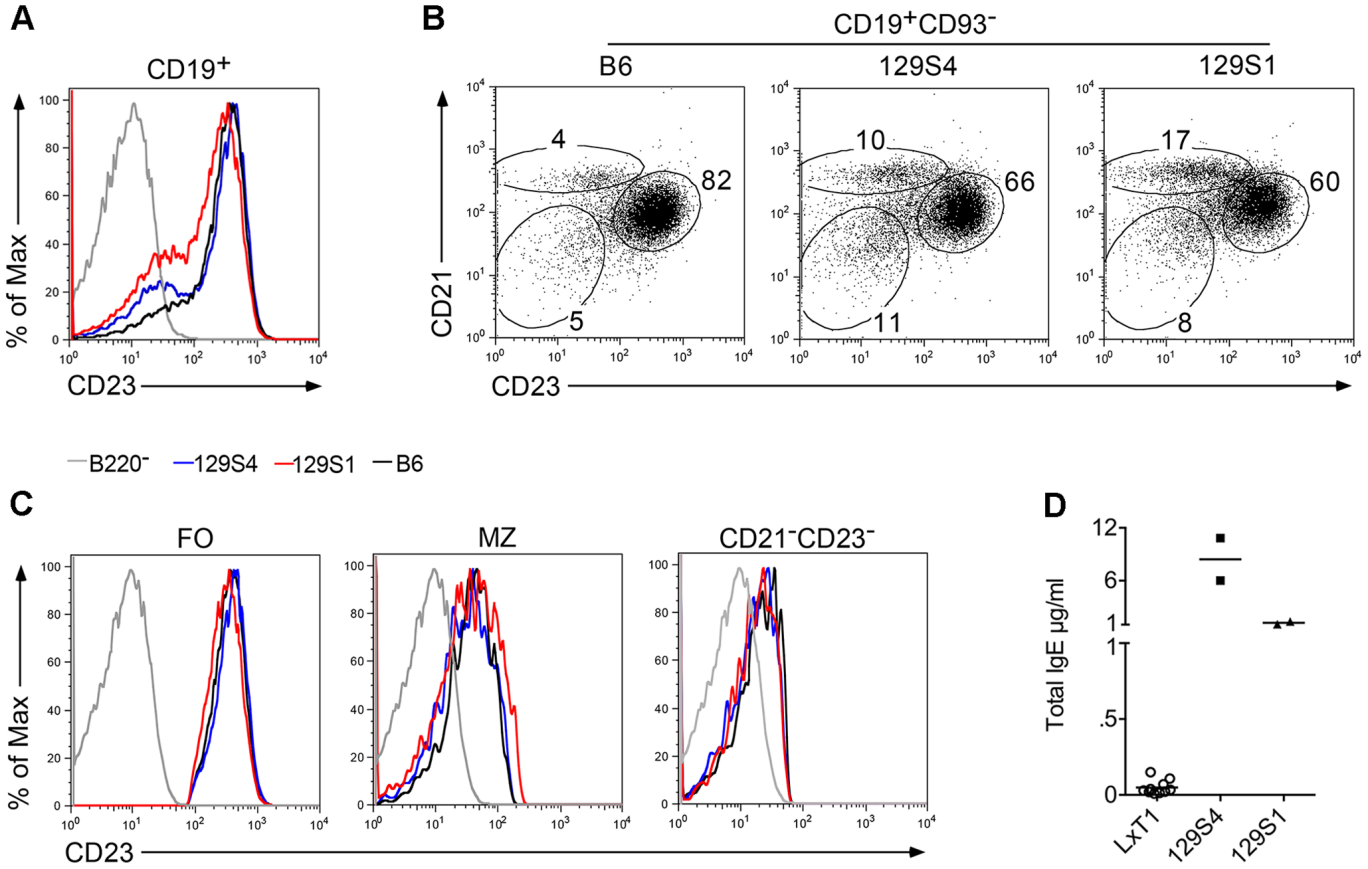
Normal CD23 surface levels and hyper IgE in 129S4 and 129S1 mice. Flow cytometry analysis of spleen cells from nine months old B6, 129S4 and129S1 mice. (A) Gating on CD19^+^ splenic B cells, graph shows CD23^low^ surface levels are not observed on B cells in 129S4 and 129S1 mice compared to B6, with a different proportion of CD23^low/−^ among these three strains. (B) Gating on mature B cells (CD19^+^CD93^−^), dot plots show increased MZ (CD21^hi^CD23^low/−^) and CD21^−^CD23^−^ B cell populations and decreased FO B cells (CD21^hi^CD23^hi^) in 129S4 and 129S1 compared to B6. (C) Within each population gated in Fig. 4B, CD23 surface levels are expressed similarly in 129S4, 129S1 and B6 mice. (D) Serum IgE levels are higher in the two 129 substrains compared to LxT1 mice. LxT1 mice were three to nine months old, while 129S4 and 129S1 substrains were nine months old.

To determine whether the CD23 mutations were directly related to the CD23^low^ surface levels, spleen cells from the 129S4 and 129S1 substrains, carrying the same five CD23 mutations as LxT1, were stained and analyzed by flow cytometry. To our surprise, the CD23 expression pattern, after gating on CD19^+^ splenic B cells, was rather similar in 129S4 and 129S1 when compared to that in B6 mice, with the majority of the cells expressing high CD23 levels and only a small proportion being CD23^low/−^ in all three strains (Fig. 4A). Gating on mature (CD19^+^CD93^−^) B cells, distinguished FO, MZ and B1 and/or age-associated B cells (CD21^−^23^−^) [23], [24] in all three strains, although the proportions differed (Fig. 4B). Nevertheless, gating on each of these B cell populations, demonstrated the same CD23 surface levels in the 129S4 and 129S1 substrains as in B6 mice (Fig. 4C). In addition, the CD23 expression pattern in the different strains was confirmed by staining with the 2G8 clone (data not shown). Since the 129 substrains carry the five CD23 mutations, these results suggest that the CD23^low^ phenotype cannot be interpreted as a direct consequence of the mutations in the CD23 coding region.

To investigate the association of the CD23 mutations and natural IgE production, total serum IgE levels in naive LxT1 and 129 substrains were compared. As shown in the Fig. 4D, serum IgE levels were substantially higher in the two 129 substrains than in LxT1 mice. The total IgE levels in B6 and LxT1 mice were less than 0.1 µg/ml (Fig. 4D and Fig. 3), whereas serum IgE concentrations in 129 mice ranged from 2 µg/ml to nearly 8 µg/ml (Fig. 4D). The fact that LxT1 and 129 mice carry the five CD23 mutations but present with different IgE levels rules out an association between natural IgE levels and the CD23 mutations.

### Decreased Intracellular CD23 Levels but Normal CD23 and ADAM10 mRNA Expression

**Figure 5:**
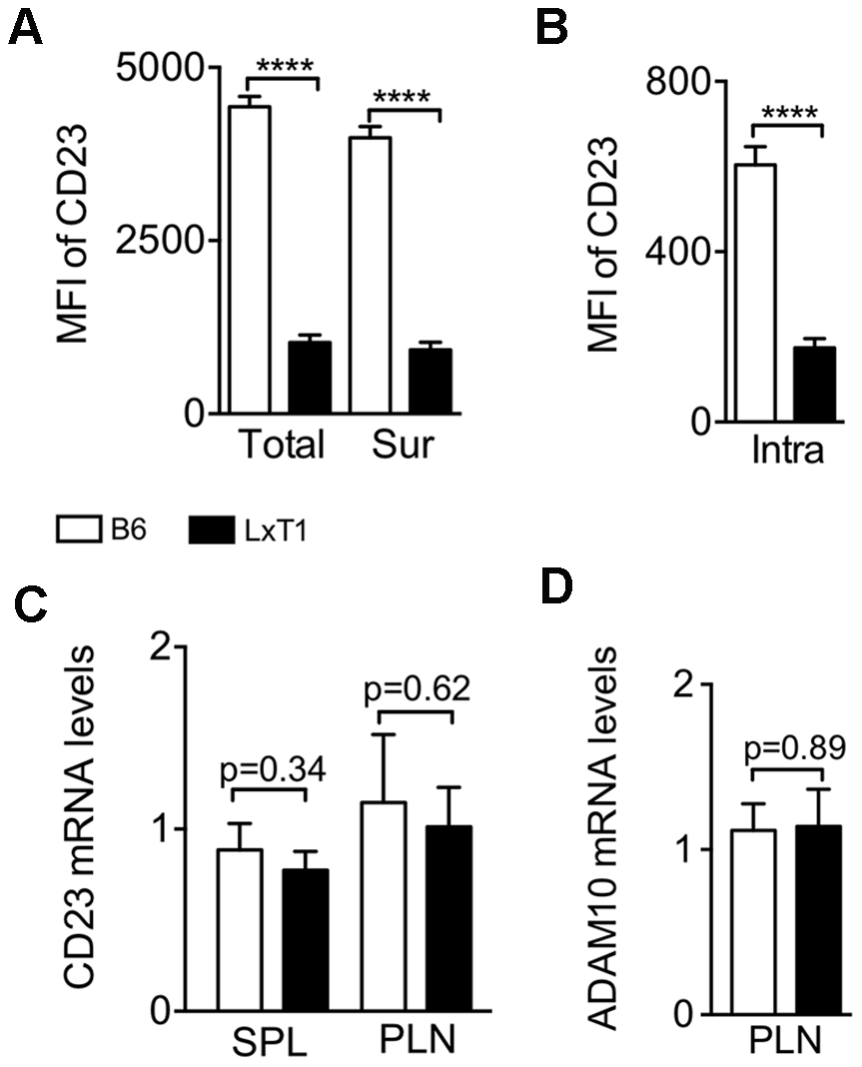
Decreased intracellular CD23 level and normal CD23 and ADAM10 mRNA transcripts. The CD23 total (Total) protein pool in LxT1 B cells was lower than that of the control (A, left), with a similar reduction in CD23 cell surface only (Sur) (A, right) and intracellular (intra) levels (B). Representative of one experiment, n = 3/genotype. (C) Comparable CD23 mRNA levels in B cells from LxT1 and B6 mice. (D) Similar levels of ADAM10 mRNA expression in PLN B cells from LxT1 and B6 mice. RNA isolated from CD19^+^ splenic (SPL) and peripheral lymph node (PLN) B cells was analyzed by q-PCR. CD23 and ADAM10 transcripts were normalized to 18S rRNA. Representative of one experiment, n = 3/genotype.

LxT1 and 129 substrains carry the same five mutations, but express different CD23 surface levels (Fig. 1D and Fig. 4A), suggesting that the CD23^low^ phenotype is not biased by modulation of a single epitope due to the mutations. However, the reduced CD23 surface expression in LxT1 B cells could be due to altered intracellular accumulation of CD23 protein. To address this possibility, staining for CD23 was performed before and after membrane permeabilization to detect the protein on the cell surface and intracellularly. Compared to the B6 control, B cells from LxT1 mice displayed lower CD23 total (cell surface and intracellular) and surface levels (Fig. 5A), which were reflected in a similar reduction in intracellular CD23 level (Fig. 5B). Thus, B cells from LxT1 mice express reduced levels of CD23 on the cell surface as well as intracellularly.

To determine whether the CD23^low^ expression is reflected at the mRNA level, we enriched for CD19^+^ splenic (SPL) and peripheral lymph node (PLN) B cells and analyzed mRNA expression from these by q-PCR. CD23 mRNA levels were similar comparing B cells from LxT1 and B6 mice (Fig. 5C), indicating that the CD23^low^ surface levels in LxT1 B cells were not as a result of a change in the level of CD23 mRNA. It has been shown that CD23 contains two cleavage sites and is cleaved from the cell surface by the metalloproteinase ADAM10, thereby releasing soluble CD23 [18]. To explore the possibility that more membrane-bound CD23 is cleaved from LxT1 B cells due to an increase in ADAM10 expression, its mRNA levels in CD19^+^ PLN B cells were analyzed. The results showed that the levels of ADAM10 mRNA were similar B cells from LxT1 and B6 mice (Fig. 5D). Thus, the levels of CD23 and ADAM10 mRNA in LxT1 B cells are about the same as those in B6 mice.

### A Recessive Mutation is Linked to the CD23^low^ Phenotype in LxT1 Mice

**Figure 6:**
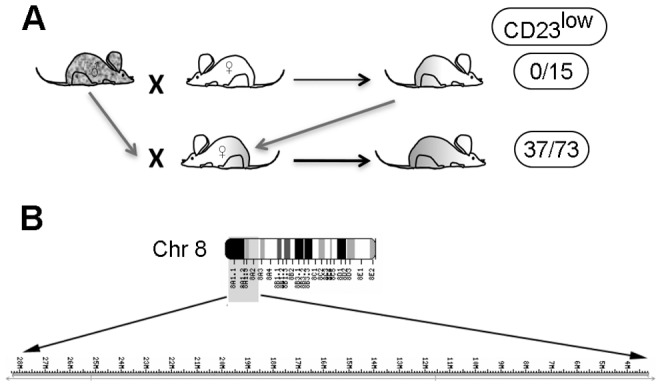
CD23^low^ surface levels in LxT1 mice due to a recessive mutation located on chromosome 8. (A) Male LxT1 mice were crossed to female BALB/c mice to generate F1 mice, from which females were backcrossed to the male LxT1 to generate offspring for analysis of CD23 phenotypes and DNA for genetic mapping. (B) The region of chromosome 8 (0 to 28.309.058) corresponds to the CD23^low^ phenotype observed in LxT1 mice based on the genetic linkage analysis.

The CD23^low^ phenotype in LxT1 mice was apparently not due to differences in epitopes, transcription, intracellular accumulation or mutations in the CD23 coding region but rather to some other genetic character. To investigate potential genetic alterations and map a responsible locus, we crossed male LxT1 (for simplicity termed LxT1^m/m^) mice to female mice of the BALB/c strain that do not display reduced CD23 surface levels and do not carry the five mutations (LxT1^B/B^). Since the LxT1 B cell phenotype could be monitored using blood (Fig. 1G), 15 offspring (LxT1^B/m^) from this first cross were analyzed by flow cytometry. All of these showed normal (B6, BALB/c) CD23 surface levels on B cells, indicating that a recessive genetic alteration is associated with the CD23^low^ phenotype in LxT1 mice. To further validate this hypothesis, female offspring from this first cross (LxT1^B/m^) were backcrossed to the male LxT1 (LxT1^m/m^) in order to obtain offspring with a genetic identity closer to LxT1, and potentially giving rise to LxT1^m/m^ and LxT1^B/m^ offspring in a ratio of approximately 1∶1. In this backcross, seventy-three mice in total were analyzed. Mice with and without a CD23^low^ phenotype were almost equal (36 and 37, respectively) in numbers (Fig. 6A), demonstrating that a recessive genetic alteration is linked to the CD23^low^ phenotype on LxT1 B cells.

### A 28 Mb Region of Chromosome 8 is Linked to CD23^low^ Phenotype in LxT1 Mice

To map a potential locus corresponding to the CD23^low^ phenotype, DNA was prepared from 11 backcrossed mice, with and without a CD23^low^ phenotype, and one BALB/c mouse as a control, and subsequently genotyped using the Affymetrix Mouse Diversity Genotyping Array. To identify the genetic linkage to the phenotype, only markers complying with the inheritance pattern were considered which correlated to 799 SNP markers. In the region of chromosome 8∶3.160.572–25.982.430, 796 SNP markers complied with the inheritance pattern. Another two markers complying with this pattern were positioned at chromosome 8∶104.653.028 and 128.699.258, which is separated by 3.447 non-complying markers from position 25.982.430. In one of the heterozygous mice, the defining crossover event occurred between the positions 25.982.430 and 28.309.058 on chromosome 8, which made the SNP at position 28.309.058 complying with a different inheritance pattern. By considering bordering markers to the candidate region, which did not comply with the pattern, the outer limits of the region were determined to be chromosome 8∶0 to 28.309.058. These results show that a 28-megabase pairs (Mb) candidate region on chromosome 8 is linked to the CD23^low^ surface levels observed in the LxT1 mice (Fig. 6B). The region is gene rich and contains CD23 in addition to another 328 genes of which many are involved in immunological functions, e.g. DC-SIGN and Shcbp1 etc. Comparing the complied SNP markers in this region with other strains, we found that the genotype of LxT1 mice follows the genotype of the 129 strain indicating that the 28 Mb region in LxT1 mice is inherited from the 129 strain during the meioses process of crossing and backcrossing.

## Discussion

In this study, we unexpectedly detected reduced surface levels of CD23 on B cells in a mouse line, LxT1, indicative of a block at the transition from the T1 to T2 stage during B cell development. However, mature B cells still develop, B cell numbers are similar to those in B6 mice. Since splenic B cells from these mice showed no significant difference in the expression of B220, CD19, CD93, IgM, IgD or CD21, additional markers that are used to define different B cell populations, i.e. T1, T2/3, MZ and FO [20], it appears that only the CD23 surface expression is affected rather than the development of different B cell populations. This reduction in CD23 surface expression is linked to a recessive genetic alteration in a 28 Mb region on chromosome 8, as demonstrated by genetic crossing and mapping. This region is derived from the 129 background and covers the CD23 gene including five mutations in its coding region that are also present in other strains, eg NZB and 129 but not B6 or BALB/c strains (Table 1). Mice with these five mutations have been reported to express reduced CD23 surface levels on B cells [11], [12], although not observed in all 129 substrains [14], a discrepancy that was interpreted as a possible artifact due to non-specific binding of the anti-CD23 antibodies [12]. Herein, we demonstrate a CD23^low^ phenotype is not found on B cells from the 129 substrains even though all carry the five CD23 mutations (Fig. 4A). Although we observed a relatively high proportion of MZ and B1/ABC cells in the 129 substrains that are CD23^−^ B cells (Fig. 4B), the levels on FO B cells was no different to those from B6 mice (Fig. 4C). Hence, some of the discrepancies between different studies may be due to the proportion of CD23 negative, i.e. MZ, B1 and ABC, B cell populations.

The NZB strain also carries the five mutations in CD23 whereas only one of these is present in NZW mice (Table 1). Crossing these two strains, results in a CD23^low^ phenotype, as in the NZB strain and it was, therefore, proposed that the CD23 mutations are dominant [11]. In our study, crossing the LxT1 to BALB/c mice suggests a recessive effect leading to the CD23^low^ phenotype. One explanation for the discrepancy could be the lack of CD23 mutations in BALB/c mice and the presence of one mutation in NZW mice.

Our data indicate that the CD23^low^ surface levels on LxT1 B cells is not due to an inability of the antibody to recognize the protein or intracellular accumulation of CD23. However, we found that the CD23 mRNA levels were similar to those in control B cells. This could indicate an increase in soluble CD23 cleaved from the cell surface as a result of elevated shedding by proteases, e.g. ADAM10 [18]. However, expression of ADAM10 transcripts was similar in B cells from LxT1 and controls, indicating the CD23^low^ surface levels on LxT1 B cells were not regulated by an increase in ADAM10 expression. Rather, because of the similarities between LxT1 and both NZB and 129/SvJ mice in terms of CD23, and because these latter strains present with reduced serum levels of soluble CD23 [11], [12], it is likely that the soluble CD23 levels are also reduced in LxT1 mice. Similar to the LxT1 mice, NZB and 129/SvJ mice carry the same CD23 mutations, express lower surface CD23 levels and, as analyzed in 129/SvJ mice, CD23 mRNA levels similar to those of control B cells. If so, a reduction in soluble CD23 would be a reflection of decreased surface and intracellular levels. Moreover, even though the mutations in CD23 may interfere with the stability of CD23 [11], [12], our data would argue that it is not sufficient to affect the CD23 surface levels on B cells. Rather, because the strains that present with the CD23^low^ phenotype have a mixed genetic background [11], [25], it is likely that the different CD23 phenotypes are also associated with genetic background, like in the LxT1 mice, the 28 Mb interval on chromosome 8. In addition to inherent complexity of CD23 biology, the genetic variation could for instance affect translation and/or internal degradation of CD23 [12], as well as other immune influences [12], [14], [26]–[28].

CD23 has been proposed to play an important role in the negative regulation of IgE levels [8], and the CD23 mutations may influence serum IgE levels: 129 mice present with higher IgE levels compared to those of the B6 and BALB/c strains [27], [29]. However, as we found that LxT1 mice display similarly low levels as B6 mice, this argues that natural IgE production and CD23 surface levels are uncoupled, and that neither is affected by the CD23 mutations *per se*. The role of CD23 in regulating IgE may rather depend on the genetic mouse models used. For instance, CD23-deficient mice on a mixed BALB/c×129 background do not produce increased IgE levels either after antigen-alum immunizations or in parasitic infection models [30], [31]. By contrast, mice with the same deficiency on a B6 background display higher IgE levels than those of controls after sensitization with antigen-alum, but reach similar levels after infection with parasites [8]. Taken together, since embryonic stem cells from 129 strains have been widely used to generate targeted and transgenic models, this highlights the importance of genetic background in the interpretation of the immunological data generated from genetically manipulated mice. This does not only apply to CD23 surface expression and serum IgE levels [11], [12], but also to other, for instance, autoimmune traits [32].
